# Bridging Citizen Science and Expert Surveys in urban biodiversity monitoring: Insights from insect diversity in Macao

**DOI:** 10.3897/BDJ.13.e153402

**Published:** 2025-06-12

**Authors:** Kaiyun Zheng, Mark K. L. Wong, Toby P. N. Tsang, Chi Man Leong

**Affiliations:** 1 Department of Life Sciences, Beijing Normal-Hong Kong Baptist University, Zhuhai, China Department of Life Sciences, Beijing Normal-Hong Kong Baptist University Zhuhai China; 2 School of Biological Sciences, The University of Western Australia, Crawley, Australia School of Biological Sciences, The University of Western Australia Crawley Australia; 3 Department of Biological Sciences, University of Toronto Scarborough, Toronto, Canada Department of Biological Sciences, University of Toronto Scarborough Toronto Canada; 4 Guangdong Provincial/Zhuhai Key Laboratory of Interdisciplinary Research and Application for Data Science, Beijing Normal-Hong Kong Baptist University, Zhuhai, China Guangdong Provincial/Zhuhai Key Laboratory of Interdisciplinary Research and Application for Data Science, Beijing Normal-Hong Kong Baptist University Zhuhai China

**Keywords:** citizen science, iNaturalist, conservation, biodiversity, urban ecosystems

## Abstract

Urban ecosystems present unique challenges for biodiversity monitoring, demanding efficient methods to document species diversity in rapidly changing environments. This study quantifies insect diversity in Macao SAR — a hyper-urbanised region — by integrating data on 1,339 species documented in expert-led surveys and 1,012 species recorded in citizen-science observations between 2019 and 2023. Striking divergence emerged between the expert and citizen-science datasets: only 462 species (33.5% of total diversity) were detected by both groups, with experts documenting 877 unique taxa often requiring specialised collection or morphological analysis, while citizen scientists contributed 550 distinctive species through spatially explicit, image-based records. Together, these approaches achieved 96.59% estimated species coverage within five years, demonstrating that combining community-driven data with expert methods accelerates comprehensive biodiversity documentation. Citizen-science platforms played a pivotal role by providing high-resolution geotagged imagery which enabled experts to validate records and resolve taxonomic ambiguities. Meanwhile, expert surveys detected cryptic taxa overlooked by citizen scientists. The rapid species coverage achieved through this synergy highlights the transformative potential of integrated frameworks. By mobilizing the scalability of citizen science to fill spatial and taxonomic gaps, while leveraging expert precision to ensure rigour, urban biodiversity monitoring can adapt to the rapid pace of ecological change. These findings advocate for collaborative strategies that harness public participation and scientific validation to optimise conservation efforts in data-deficient and highly-stressed ecosystems.

## Introduction

With the acceleration of global urbanisation, cities are increasingly recognised not only as centres of human activity, but also as complex, dynamic ecosystems that harbour significant biodiversity. Biodiversity studies are essential for understanding how species respond to environmental changes and for developing effective strategies for natural resource management and ecosystem sustainability (Savard et al. 2000, Uchida et al. 2021). Urban biodiversity plays a particularly critical role in sustaining ecosystem services— such as air and water purification, climate regulation and recreational opportunities —that directly enhance the quality of life for city dwellers (Faeth et al. 2011, Shwartz et al. 2014). Moreover, urban ecosystems serve as valuable ecological models, providing a natural laboratory to study species adaptations under varying levels of anthropogenic stress (Schwarz et al. 2017, Lambert and Donihue 2020).

Traditionally, expert-led surveys — employing systematic methods like plot sampling, transect walks and expedition trips — have been the cornerstone of biodiversity monitoring (Oertli et al. 2005, Hill 2005, Magurran and McGill 2011). While these methods offer high taxonomic precision and robust data quality, they are often constrained by limited resources, spatial and temporal coverage and the logistical challenges of sampling fragmented urban landscapes. In recent years, citizen science (CS) has emerged as a transformative tool that complements traditional survey methods by engaging the public through digital platforms, such as iNaturalist (Bonney et al. 2014, Pocock et al. 2017). Urban environments, with their high population densities and accessible green spaces, have proven particularly conducive to CS initiatives (Li et al. 2019, Callaghan et al. 2020). The integration of artificial intelligence and digital tools has further streamlined data collection and species identification (Farley et al. 2018, Vohland et al. 2021). As a result, CS projects have contributed nearly 640 million biodiversity records globally, providing crucial insights into species distributions, invasive species detection, checklist development and even new species discoveries (Chandler et al. 2017, Freitag et al. 2018, Fagan-Jeffries and Austin 2021).

Despite its promise, CS is not without challenges. Significant biases persist in the geographic and taxonomic coverage of CS data, often reflecting a disparity between urban and natural habitats (Chandler et al. 2017, Sheard et al. 2020, Díaz-Calafat et al. 2024). Studies show that citizen scientists tend to record species with larger body sizes or prominent external morphological features — attributes that facilitate visual identification via smartphones — leading to an observational preference that may overlook smaller, cryptic taxa (Aristeidou et al. 2021, Randler et al. 2023, Alfeus et al. 2024). Moreover, while CS rapidly accumulates data and can achieve near-complete species coverage in a short time, this saturation means that, after several years, the discovery of new species slows considerably. Nonetheless, as the bulk of CS biodiversity data has originated from Western countries (Hughes et al. 2021), it is unclear if such trends also characterise CS biodiversity monitoring efforts in other regions globally. Moreover, it remains unclear how CS observations of regional biodiversity compare to those obtained from expert-led surveys and if the former indeed complement the latter as is generally assumed.

To understand how CS compares with expert-led surveys and contributes to biodiversity monitoring in an urban, non-Western context, we focused on Macao SAR, a city in subtropical Asia which houses one of the world’s densest human populations in a highly urban landscape (Tang and Sheng 2009). Despite high urbanisation, Macao supports considerable biodiversity, especially for insects (Brassard et al. 2021). We compared patterns of regional insect diversity derived from traditional expert-led surveys with those obtained from a CS database (iNaturalist). We predicted that the CS approach would rapidly accumulate species records and approach near-complete sampling coverage for many insect orders, but that its detection rate would plateau over time, yielding fewer new records. In contrast, expert-led surveys, with their specimen-based methods, would be more effective in documenting less conspicuous species. By comparing these two approaches, our study highlights their complementary roles in urban biodiversity monitoring. An integrative framework combining CS and expert surveys can enhance our understanding of urban insect assemblages and provide information for conservation strategies for sustaining urban ecosystems. Ultimately, we advocate for the combined use of both methods to achieve a more comprehensive and accurate assessment of biodiversity in rapidly urbanising areas.

## Material and methods

**Data Collection**

We compiled two comprehensive, but distinct datasets of all insect species occurrences within Macao SAR: a CS dataset and an expert dataset. To compile the CS dataset, CS data were obtained from the iNaturalist platform (www.inaturalist.org), which facilitates public participation in biodiversity monitoring. We extracted all records for the class Insecta within Macao — defined by the geographic bounds 22.1068°N to 22.2335°N and 113.5367°E to 113.5653°E — including all observations recorded up to 31 December 2023. To ensure data quality, we filtered the dataset to include only research-grade observations (i.e. records with majority agreement on identifications and accompanied by photographs, precise georeferences and observation dates). The expert dataset was compiled from a comprehensive expert-derived insect checklist for Macao, assembled from handbooks, monographs, published articles and official records (Xian et al. 2024). Xian et al. (2024) conducted a comprehensive review of all available published records up to 2023, while our study extends this effort by incorporating CS data up to the same year to enable comparative analyses and a more comprehensive assessment of urban insect diversity in Macao. This literature-based dataset represents a formal, taxonomically verified account of insect species in the region. Together, these two data sources provide a robust foundation for comparing species richness and composition across 14 insect orders (Blattodea, Coleoptera, Dermaptera, Diptera, Embioptera, Hemiptera, Hymenoptera, Lepidoptera, Mantodea, Neuroptera, Odonata, Orthoptera, Phasmida and Zygentoma), thereby enabling a critical evaluation of the effectiveness and limitations of CS in urban biodiversity monitoring. In the subsequent data analysis, both datasets were restricted to the species level, with subspecies-level records excluded from the analysis to maintain consistency in taxonomic resolution. To ensure data consistency and accuracy, we standardised the taxonomic classifications between the two datasets, resolving potential synonymy issues and minimising discrepancies in species identification.

**Data Analysis**

To quantify insect community diversity and assess sampling trends in Macao, we applied rarefaction and species accumulation analyses to the CS dataset.

For comparative analysis, the insect species recorded via iNaturalist were consolidated into a comprehensive list and then compared with the expert checklist. Species were categorised into three groups: those exclusively documented on iNaturalist (CS dataset-only), those unique to the expert dataset (expert dataset-only) and those shared between both sources. This categorisation was performed separately for each of the 14 insect orders recorded to examine differences in total species richness and assess the extent of species overlap. To assess the distribution of species richness data across the three groups (CS dataset-only, expert dataset-only and shared species), we performed a Shapiro-Wilk test for normality using the shapiro.test() function from the *stats* package (Royston 1982, Royston 1995, R Core Team 2024). Since the data were not normally distributed, we applied a non-parametric Friedman test using the friedman.test() function from the *stats* package to compare species representation across datasets (Hollander and Wolfe 1973, R Core Team 2024).

To assess the overall trend in insect diversity across the two data sources, we conducted a linear regression analysis. To evaluate overall insect diversity trends across the two data sources, we conducted a linear regression analysis using the lm() function from the *stats* package (R Core Team 2024), each data point representing the number of species recorded for a given insect order from both datasets. The regression was calculated using the least squares method and the coefficient of determination (R²) was computed to assess the model’s goodness-of-fit (Su et al. 2012, Hoffmann 2022).

To quantify insect community diversity and assess sampling trends in Macao, we applied rarefaction and species accumulation analyses to the CS dataset. To estimate species coverage for the CS dataset, both overall and for each insect order, we employed the iNEXT model using the iNEXT() function from the *iNEXT* package (Chao et al. 2014, Hsieh et al. 2016).

All statistical analyses were performed in R (version 2023.03.0+384, R 4.2.3; R Core Team (2024)). We used the *tidyverse*, *dplyr* and *tidyr* packages to filter, summarise and transform the data, as well as to reshape the datasets into tidy formats suitable for statistical analysis and visualisation (R Core Team 2024). Data visualisation was performed using the *ggplot2* package, with additional enhancements and publication-ready formatting applied using the *ggpubr* package (Wickham 2016, Kassambara 2023).

## Results

**Overview of data sources**

**Figure 1.**
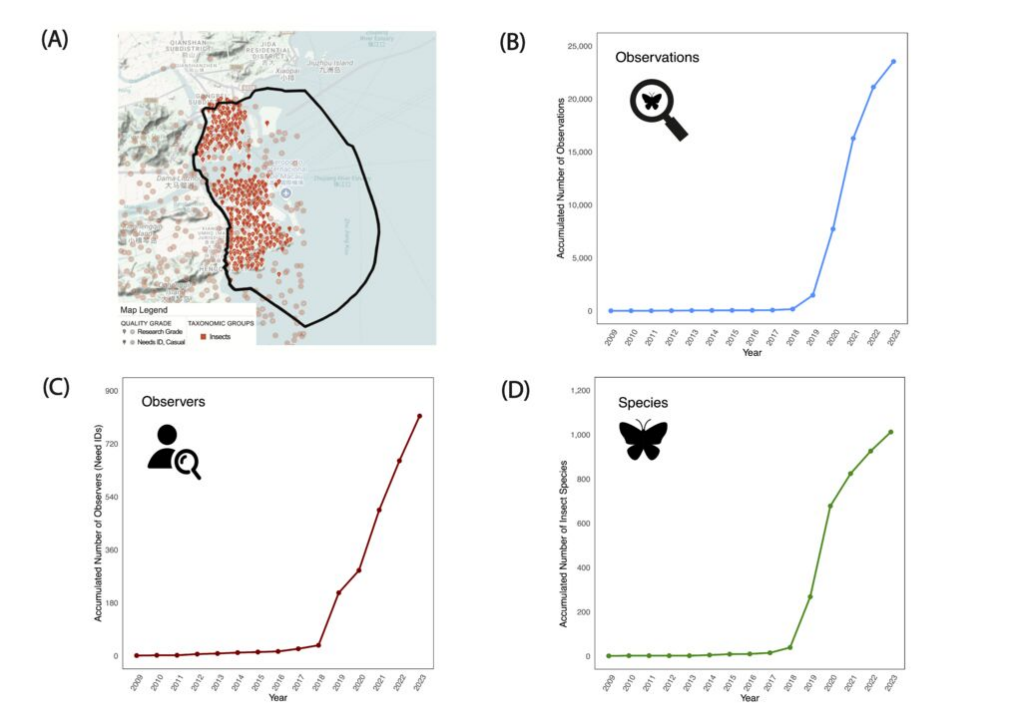
Map showing observations of insect species occurring within the boundaries of Macao (in black) in the iNaturalist.org dataset during the study period of 2010–2023 (A). Plots show cumulative trends over the study period in the number of identifiers which had both a verifiable User ID and a Research Grade identification (B), the number of observations of insects (C) and the number of insect species observed (D).

We compiled two distinct datasets to assess urban insect diversity in Macao. The CS dataset, derived from iNaturalist, comprised 23,535 observations recorded between 2010 and 2023 (Fig. 1C). These records, contributed by 814 unique observers and validated by 426 research‐grade identifiers, encompassed 1,012 insect species spanning 14 orders (Fig. 1B, D and E). In parallel, the expert dataset, assembled from handbooks, taxonomic monographs, published articles and official records (Xian et al. 2024), documented a total of 1,339 species. Together, these datasets provided a comprehensive basis for evaluating the effectiveness of CS in capturing urban insect diversity.

**Insect Diversity in iNaturalist**

**Figure 2.**
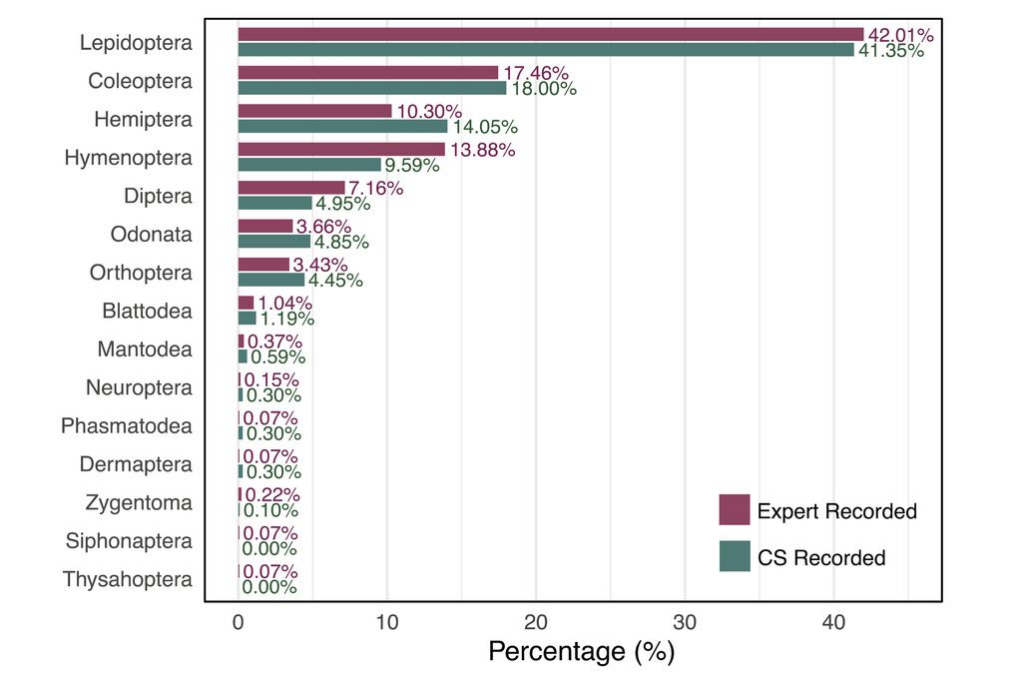
Taxonomic breakdown of insect diversity recorded in the expert dataset and the CS dataset. Bars represent the relative proportion of each insect order recorded by each dataset.

CS contributions revealed pronounced variation in species documentation across insect orders (Table 1, Fig. 2). Lepidoptera dominated records, accounting for 418 species (41.3% of total observations), followed by Coleoptera (182 species, 18.0%) and Hemiptera (142 species, 14.0%). Moderately represented groups included Hymenoptera (97 species, 9.6%), Diptera (50 species, 4.9%), Odonata (49 species, 4.8%) and Orthoptera (45 species, 4.4%). Notably under-represented taxa spanned Blattodea (12 species), Mantodea (6 species) and several orders with ≤ 3 species each: Zygentoma (1), Neuroptera (3), Phasmatodea (3), Dermaptera (3) and Embioptera (1). No records were reported for Siphonaptera or Thysanoptera.

**Comparison of citizen science and expert records of insect diversity**

To assess the effectiveness of CS versus expert-led surveys of insect biodiversity, we compared the insect species lists from iNaturalist and the expert-derived literature checklist. Table 1 details the number of species per insect order for both datasets, along with the number of shared species. For instance, Lepidoptera were represented by 565 species in the expert dataset and 418 in the CS dataset, while Coleoptera and Hemiptera showed similar patterns, albeit with slightly lower absolute species richness in the CS data. Conversely, the expert dataset documented substantially more species of Hymenoptera (185) and Diptera (94) compared to the CS records (97 and 50, respectively). The percentage of shared species — calculated as the proportion of species common to both sources —varied by taxonomic order; several orders, such as Odonata, exhibited high overlap amongst datasets (67%), whereas others, such as Phasmatodea and Hymenoptera, had substantially fewer shared species. Across all orders, the average proportion of species common to both datasets was approximately 29% for the expert dataset and 28% for the CS dataset, indicating a moderate level of concordance. Since the data for all three groups — CS, expert (E) and shared species richness —were not normally distributed (Shapiro-Wilk test, p < 0.001), we used the non-parametric Friedman test to compare them. The Friedman test results (χ² = 20.237, df = 2, p < 0.001) indicate significant differences in species representation amongst the three groups, suggesting systematic variation in how insect biodiversity is documented across datasets.

**Linear regression analysis of insect orders**

**Figure 3.**
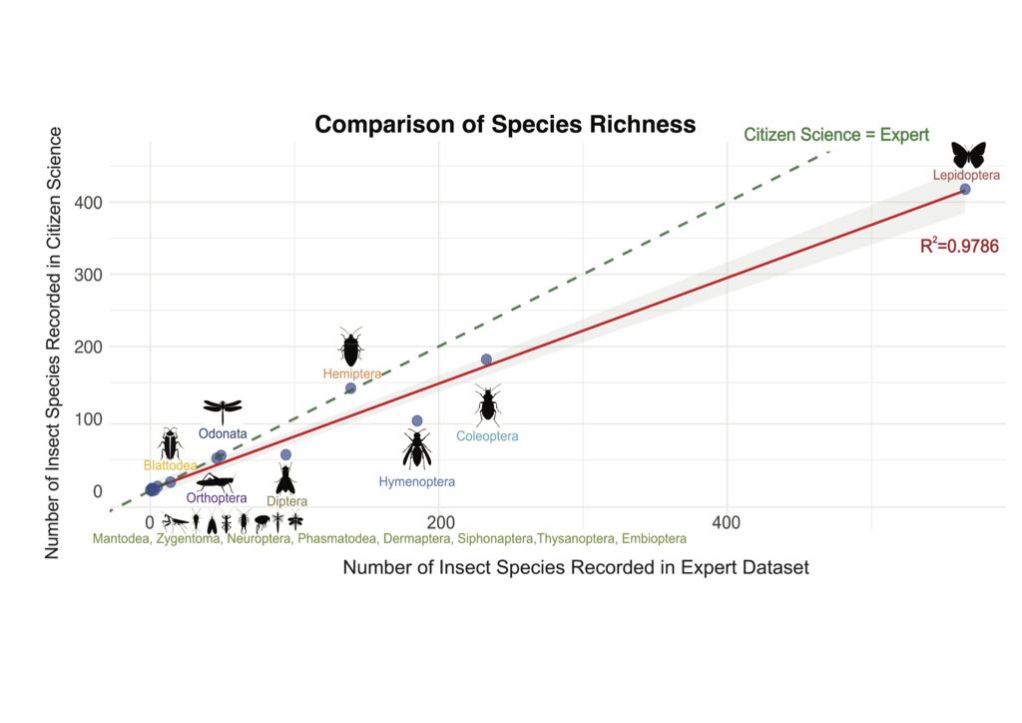
Linear regression between values of insect species richness in the CS and expert datasets. The x-axis represents the number of species documented in expert datasets, while the y-axis shows the number of species recorded in the CS dataset. Each point represents an insect order, labelled accordingly. The solid red line is the regression line, showing the overall relationship between the two datasets and the dashed green line represents the 1:1 line, where species richness would be equal between the two sources.

To assess whether CS data reliably capture overall insect diversity patterns, we performed a linear regression analysis comparing the number of species recorded per insect order by CS and expert surveys (Fig. 3). Since diversity patterns can vary across insect orders, our analysis accounts for these taxonomic differences. The results revealed a strong positive correlation (R² = 0.9786, p < 0.001), with a regression slope of 0.733 (SE = 0.029). This indicates that, while CS data broadly reflect expert-documented species richness trends, the slope being significantly less than 1 suggests that CS tends to under-represent species richness relative to expert surveys. Despite some discrepancies in absolute species counts for specific orders, these findings suggest that CS provides a reliable approximation of insect diversity patterns in Macao.

**Species Coverage and Temporal Trends**

**Figure 4.**
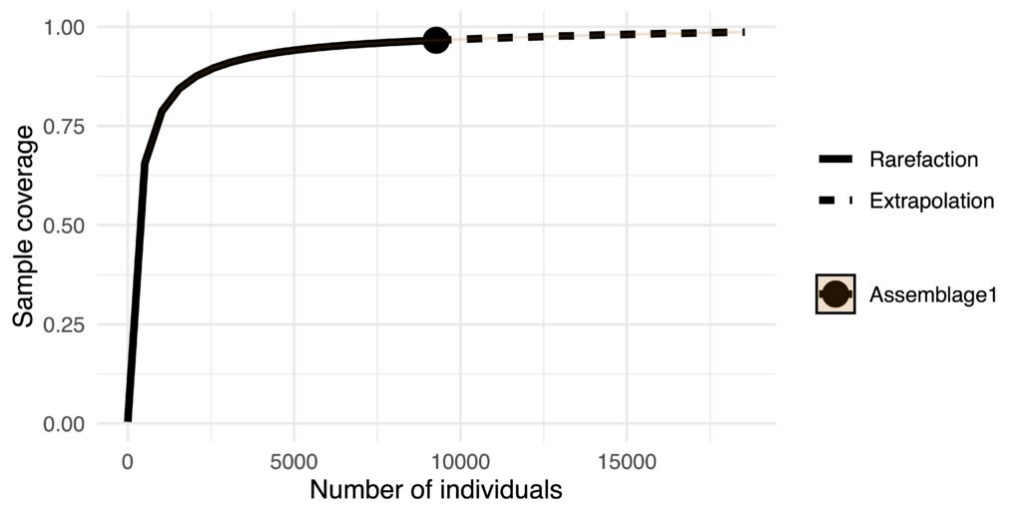
Sample completeness curve of all insect orders recorded in the CS dataset. The dashed line represents extrapolation, estimating the coverage beyond the observed data.

**Figure 5.**
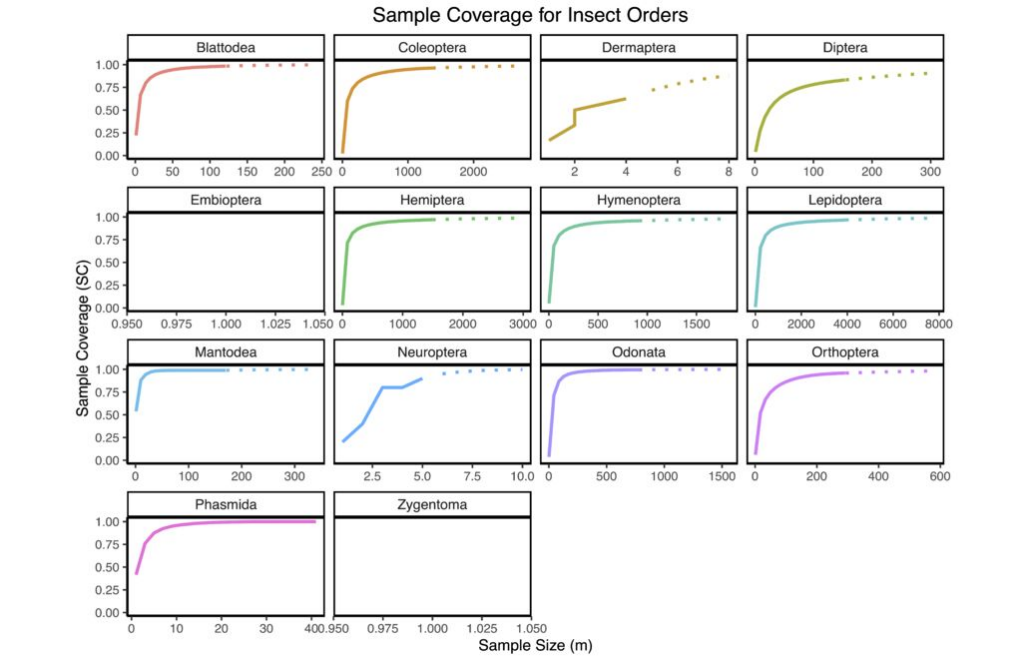
Sample completeness curves for **A** Blattodea; **B** Coleoptera; **C** Dermaptera; **D** Diptera; **E** Embioptera; **F** Hemiptera; **G** Hymenoptera; **H** Lepidoptera; **I** Mantodea; **J** Neuroptera; **K** Odonata; **L** Orthoptera; **M** Phasmida; **N** Zygentoma. Due to only a single species being observed, curves for Zygentoma and Embioptera could not be visualised.

The CS dataset achieved 96.59% estimated species coverage across all insect orders, as indicated by species accumulation and rarefaction curves (Fig. 4). Species coverage for the 14 insect orders documented in this study is detailed in Fig. 5.

## Discussion

This study provides a detailed assessment of how well CS initiatives document insect diversity in a highly-urbanised city and how they complement traditional expert-derived records. The results present a complex picture: CS data show a strong correlation with expert data in terms of insect orders and diversity (R² = 0.9786, Fig. 3) and exhibit high sampling completeness (96.59%). However, significant taxonomic discrepancies remain, indicating differences in how certain insect groups are recorded across the two datasets. These findings have important implications for urban biodiversity research and management, emphasising both the strengths and limitations of CS in ecological monitoring.

First, the rapid accumulation of CS data — evidenced by the marked increase in observations and species richness post-2018 in Macao (Fig. 1) — illustrates the remarkable capacity of CS to document urban biodiversity. The ease of data collection through smartphone cameras and online platforms allows for real-time, large-scale monitoring that is both cost-effective and extensive in spatial and temporal coverage (Bonney et al. 2014, Pocock et al. 2017). This high level of public engagement is particularly crucial in urban areas like Macao, where the density of observers can compensate for the challenges posed by fragmented habitats and limited access. The ability of CS to achieve near-saturation in species records, as shown by the rarefaction curves (Figs 4, 5), underscores its potential to provide a comprehensive picture of common and widespread taxa. This result aligns with previous studies, as the increasing number of observations leads to a rise in recorded species, with the extrapolated species richness in the rarefaction curve surpassing that of urbanised survey sites, highlighting the significant discovery potential of CS for common species while remaining limited for rarer or functionally important taxa (Pereira et al. 2024). This phenomenon may be related to the characteristics of the urban environment, as many species within these taxonomic groups are typically associated with artificial habitats, streetlights and other urban infrastructure, making them more easily observed and recorded by the public (Knape et al. 2022, Mandeville et al. 2022).

However, despite these strengths, our analysis also reveals inherent limitations. Notably, certain insect orders — such as Hymenoptera and Diptera — are systematically under-represented in the CS dataset compared to the expert‐derived records. This disparity likely stems from several factors. First, smaller and less conspicuous species are inherently more challenging for non-specialists to detect and identify, leading to a bias towards larger, more visually striking organisms like many Lepidoptera (McGeoch 1998, Silvertown 2009). Second, the reliance on photographic records without specimen collection restricts the capacity for detailed taxonomic verification, which can result in lower species richness for groups that require careful morphological examination (Koch et al. 2023, Tiago et al. 2024). These limitations highlight the need for integrative approaches that combine the broad reach of CS with the taxonomic rigour of expert surveys.

Our analysis of species diversity by insect order (Table 1) further illuminates the nuances in data collection. While the CS data robustly documented insects of multiple orders such as Lepidoptera, Coleoptera and Hemiptera — and even contained relatively more records of Coleoptera and Hemiptera — the observed levels of species richness were still lower than those reported in expert datasets. This suggests that CS excels in capturing trends and patterns in urban biodiversity, but could overlook some species such as rare and cryptic species that are more likely to be detected through targeted, expert-led sampling. The high proportion of shared species in orders like Lepidoptera indicates that, for some taxa, CS and expert surveys are highly complementary. Yet, in groups with lower shared proportions, the gap between public observation and systematic expert recording becomes evident, particularly in cases where public observations contribute many unique records or, conversely, where species documented by citizen scientists have already been extensively recorded in systematic surveys. The linear regression analysis (Fig. 3) corroborates the overall consistency between the two datasets and this pattern shows that, while CS is a powerful platform for documenting insect species, it should ideally be used in conjunction with expert methods to achieve a complete understanding of urban biodiversity.

From an applied ecology perspective, the implications of our findings extend to urban conservation and management. The high sampling coverage and rapid data accumulation achieved by CS platforms have the potential to revolutionise urban biodiversity monitoring. For instance, the extensive spatial and temporal data provided by CS can provide information for urban planning initiatives by identifying biodiversity hotspots and areas where invasive species are emerging (Johnson et al. 2020, Encarnação et al. 2021). Moreover, the integration of CS with traditional expert surveys can enhance the accuracy of biodiversity assessments, thereby supporting evidence-based conservation policies and adaptive management strategies in rapidly urbanising regions (Grimm et al. 2008, Lee et al. 2021). Our study also highlights the importance of technological advancements in improving CS data quality. Innovations in automated species identification, facilitated by machine-learning and artificial intelligence, have the potential to mitigate some of the observer biases inherent in CS datasets (Farley et al. 2018, Hecker et al. 2018).

In addition, targeted training programmes for citizen scientists could enhance their ability to detect and accurately record under-represented taxa, such as Hymenoptera and Diptera (Johnson et al. 2014, Díaz-Calafat et al. 2024). By combining these technological and educational strategies with the extensive data collection capabilities of CS, future monitoring programmes could yield even more comprehensive insights into urban insect diversity. Beyond Macao, our results have broader implications for urban biodiversity management in other cities facing similar challenges — such as New York, Hong Kong and Singapore — where rapid urbanisation, fragmented habitats and resource constraints make traditional biodiversity surveys difficult to implement. In such contexts, integrating CS with expert data can bridge critical knowledge gaps, as the CS dataset recorded 877 unique species not found in expert surveys (Table 1), demonstrating its value in capturing overlooked biodiversity. While validation is necessary to ensure accuracy, combining both data sources offers a cost-effective and efficient means of tracking biodiversity trends, while maximising taxonomic coverage and sampling breadth. This hybrid approach not only enhances the reliability of species inventories, but also empowers communities to participate actively in conservation efforts, fostering a culture of environmental stewardship that is essential for long-term urban sustainability (Sheard et al. 2020, Callaghan et al. 2021).

In conclusion, while CS data have some limitations — particularly in detecting rare or cryptic species — they serve as an invaluable complement to expert surveys in urban biodiversity monitoring. Our study highlights this by documenting a rare species *Mortonagrionhirosei* (Morimoto et al. 2010), a Near Threatened species on the IUCN Red List, demonstrating the potential of CS to contribute meaningful records for conservation. The integration of these methods can provide a more comprehensive understanding of species distributions and community dynamics in urban ecosystems. Future research should focus on refining data integration techniques, improving automated identification tools and enhancing training for citizen scientists. Such efforts will not only bolster the accuracy of urban biodiversity assessments, but also support the development of adaptive, evidence-based conservation policies that are critical in the face of ongoing urbanisation and environmental change.

## Figures and Tables

**Table 1.** Comparison of insect species records in the CS and expert datasets. The table presents the number of species recorded for each insect order in both CS and expert datasets, as well as the number of shared species between the two datasets. The columns "% Shared (Expert = E)" and "% Shared (Citizen Science = CS)" represent the proportion of shared species out of the total recorded species for each order in expert and CS datasets, respectively.

**Order**	**E**	**CS**	**Shared Sp**	% **Shared (E)**	% **Shared (CS)**
Lepidoptera	565	418	219	39%	52%
Coleoptera	233	182	83	36%	46%
Hymenoptera	185	97	48	26%	49%
Hemiptera	139	142	49	35%	35%
Diptera	94	50	16	17%	32%
Odonata	49	49	33	67%	67%
Orthoptera	46	45	11	24%	24%
Blattodea	14	12	5	36%	42%
Mantodea	5	6	2	40%	33%
Zygentoma	3	1	0	0%	0%
Neuroptera	2	3	1	50%	33%
Phasmatodea	1	3	1	100%	33%
Dermaptera	1	3	0	0%	0%
Siphonaptera	1	0	0	0%	0%
Thysanoptera	1	0	0	0%	0%
Embioptera	0	1	0	0%	0%
**Total**	**1,339**	**1,012**	**462**	**Avg. 29**%	**Avg. 28**%
